# Effect of observer’s cultural background and masking condition of target face on facial expression recognition for machine-learning dataset

**DOI:** 10.1371/journal.pone.0313029

**Published:** 2024-10-30

**Authors:** Masahito Nagata, Katsunori Okajima

**Affiliations:** Faculty of Environment and Information Sciences, Yokohama National University, Yokohama, Kanagawa, Japan; CNRS: Centre National de la Recherche Scientifique, FRANCE

## Abstract

Facial expression recognition (FER) is significantly influenced by the cultural background (CB) of observers and the masking conditions of the target face. This study aimed to clarify these factors’ impact on FER, particularly in machine-learning datasets, increasingly used in human-computer interaction and automated systems. We conducted an FER experiment with East Asian participants and compared the results with the FERPlus dataset, evaluated by Western raters. Our novel analysis approach focused on variability between images and participants within a "majority" category and the eye-opening rate of target faces, providing a deeper understanding of FER processes. Notable findings were differences in "fear" perception between East Asians and Westerners, with East Asians more likely to interpret "fear" as "surprise." Masking conditions significantly affected emotion categorization, with "fear" perceived by East Asians for non-masked faces interpreted as "surprise" for masked faces. Then, the emotion labels were perceived as different emotions across categories in the masking condition, rather than simply lower recognition rates or confusion as in existing studies. Additionally, "sadness" perceived by Westerners was often interpreted as "disgust" by East Asians. These results suggest that one-to-one network learning models, commonly trained using majority labels, might overlook important minority response information, potentially leading to biases in automated FER systems. In conclusion, FER dataset characteristics differ depending on the target face’s masking condition and the diversity among evaluation groups. This study highlights the need to consider these factors in machine-learning-based FER that relies on human-judged labels, to contribute to the development of more nuanced and fair automated FER systems. Our findings emphasize the novelty of our approach compared to existing studies and the importance of incorporating a broader range of human variability in FER research, setting the stage for future evaluations of machine learning classifiers on similar data.

## Introduction

The recognition of facial expressions is increasingly important in various human-computer interaction applications [1, 2], such as automatic driving technology [3, 4]. With recent advances in machine learning, particularly deep convolutional neural networks, higher precision in facial expression recognition (FER) has been achieved for non-masked faces [5–7]. However, alongside the advancements in machine learning, it is also crucial to consider the human factors that influence FER. In this study, we aim to explore the impact of the observer’s cultural background (CB) and the wearing of masks on the target face on the judgment of FER datasets. Although machine learning models are trained with data labeled by human observers, the variability in these labels due to differences in judgment groups and the physical conditions of the target face, such as masking, has not been thoroughly explored. By focusing on these aspects, our study seeks to provide insights that are not only relevant for improving machine-learning-based FER but also for enhancing our understanding of the human cognitive processes involved in facial expression recognition. Moreover, this research sets the stage for future evaluations of machine learning classifiers on similar data, addressing the need to consider these human factors in the development and evaluation of FER models.

### Machine-learning FER dataset

In this section, we illustrate that machine-learning-based facial expression recognition relies on human-labeled datasets, and these labels can vary depending on the group of evaluators. We present a specific example where the same facial images are evaluated in two different datasets, yet the emotional labels differ. FER2013 [2] is a commonly used [6] FER dataset that provides a set of non-masked facial images and emotion labels (35,885 sets) assigned by the authors and some labelers. Other researchers (FERPlus [3]) pointed out that FER2013 contained “False” labeling results. Therefore, these FERPlus researchers re-evaluated the FER2013 facial image set with data on 10 observers and released FERPlus with new labels [3]. Table 1 shows examples of emotions labeled differently between FER2013 and FERPlus. The leftmost face (#32230) was originally labeled as "surprise" by FER2013 labelers and was re-labeled as "happiness" by FERPlus labelers. This variability highlights the significance of considering human factors, such as cultural background and physical conditions like masking, in the development and evaluation of FER models. For this research, we employed the labels from the FERPlus dataset as a comparison target, as they are considered to be relatively accurate, providing a basis for further exploration into the diversity of human judgments in FER.

**Table 1:** Examples of FER labels in FER2013 [2] and FERPlus datasets [3].

Dataset	Face ID					
	#32230	#32239	#32262	#32275	#32582	#32685
FER2013	Surprise	Fear	Fear	Sadness	Surprise	Anger
FERPlus	Happiness	Anger	Sadness	Fear	Happiness	Sadness

*Note*. For copyright reasons, the dataset identification numbers (Face ID) are shown instead of the actual face images from the dataset. Therefore, these face IDs and labels do not correspond to the artificial face images in Fig 1.

This type of confusion is seen in the cases of FER datasets, as well as the general object recognition field. Curtis [8] reported that approximately 6% of images are labeled incorrectly in the ImageNet dataset [9], which consists of approximately 10 million general object images and 1,000 distinct class labels. They also reported that 10 other important datasets also contain many incorrect labels. However, in the FER field, we should consider whether judgments by the minority group should be unconditionally labeled as "False" or as diversity among different groups. Due to the diversity in today’s society, it is increasingly important for machine learning models to adapt to people from various cultural backgrounds. Therefore, in this study, as a first step in considering cultural diversity, we compared two cultural regions, East Asia and the West. To do this, we conducted a facial expression recognition (FER) experiment with participants from East Asia and compared the results with the labels provided by Western raters in the FERPlus dataset.

**Fig 1:**
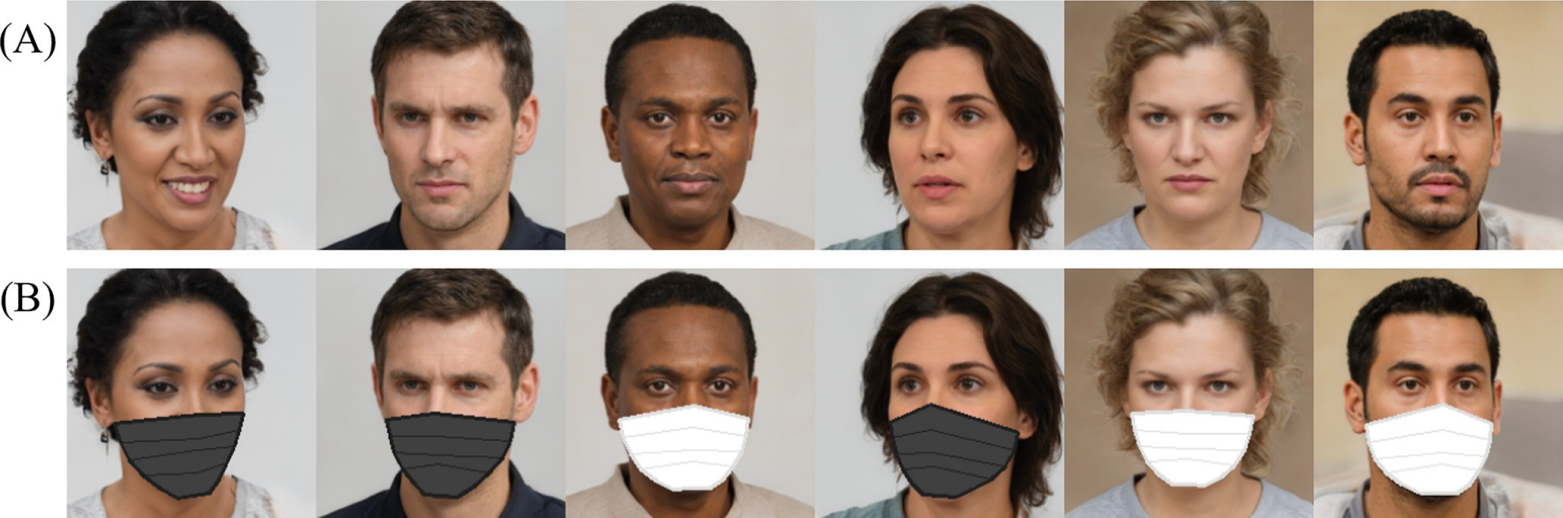
Examples of facial expressions with and without masks. (A) To satisfy the copyright policies, in this Figure, we use artificially generated faces from the website https://generated.photos, which provides unique facial portraits that do not exist. Reprinted from https://generated.photos under a CC BY license, with permission from the website’s owner, original copyright 2024. The images of the faces are examples for illustrative purposes only and are different from the original images used in the study. Therefore, these are irrelevant to Table 1. In the experiments, we used real face images from the FER2013 [2] dataset. (B) The FER2013 [2] and FERPlus [3] datasets did not contain either images or labels for the masked faces. Therefore, the original images have been processed using artificial face masks that were synthesized, as described in the Methods section.

On the other hand, the need for FER using HCI or machine learning to recognize the facial expressions of masked faces accurately has also risen owing to the increased frequency of mask-wearing in recent years. For example, in the FER of drivers in relation to automatic driving technology [6, 7], knowledge of the effect of shielding on FER is required. Fig 1 shows some facial expressions with and without masks. These comparisons show that even the expressions on masked faces (Fig 1B) could be perceived to some extent. However, the confidence in the recognition becomes lower than that for non-masked faces owing to the lack of facial information in the lower half. It remains unclear how the shielding of the target face affects FER in natural facial expressions in such real-world situations. To address this gap, our study investigates the effects of the observer’s CB and the mask shielding on the target face. We use the facial image set provided by FER2013 and the updated expression label set by FERPlus for this purpose. Regarding the two datasets FER2013 and FERPlus, it should be noted that both datasets share the same facial images originally from FER2013. The difference between the two datasets lies solely in the labeling. While we mentioned the comparison with FER2013 labels to illustrate the improvements made in FERPlus, in terms of labels, our study exclusively used the evaluation labels provided by 10 Western participants, which are considered to be more accurate.

### Previous human FER studies

In this section, we introduce previous human FER studies that focused on various factors, including the cultural background, age, and gender of the observer and the masking condition of the target face. In early human FER studies [10–12], Ekman and Friesen [10] defined six basic emotion labels, anger, disgust, fear, happiness, sadness, and surprise. Recently, seven labels (the six aforementioned labels plus neutral) have been commonly adopted as emotion labels [2, 6, 7, 13–15]. Previous studies on the cultural background (CB) differences of observers [16–18] reported that the precision of FER (the percentage of estimated labels that correctly matched the label of the facial images) differed depending on the observer groups. A previous study [19] conducted an FER evaluation experiment with 13 Westerners and 13 East Asians, focusing on the eye movements of evaluators during FER, and found that East Asians tended to focus on the eye region during emotion recognition compared to Westerners. Furthermore, the study of Fouty et al. [20] revealed statistically significant differences in the precision between different CBs. However, uncertainty remains regarding which emotions are confused across different CBs. With respect to the masking effect, previous studies have reported on the reduced precision of FER for masked faces compared to that for non-masked faces [21–26] for both adults [27–30] and children [26, 31]. Grundmann, Epstude, and Scheibe [21] found that this precision decreased from 69.9% for non-masked faces to 48.9% for masked faces for Germans. Carbon [30] reported that "disgust" on a masked face was misidentified as "anger," whereas other emotions were misidentified as "neutral." As these studies [21, 30] used the FACES dataset [32], which contains only six labeled facial emotions, thier analysis did not include "surprise," one of the seven basic emotion labels. In addition, these studies [21, 30] reported the matching rate of the masked condition as the percentage of agreement of labels between the masked condition by their experimental participants and the non-masked condition by the FACES dataset labelers, under the assumption that the FACES label is "True." That is, different groups evaluated the masked and non-masked conditions. To compare the effects of face masks, the same target faces in both the masked and non-masked conditions should be observed by people in the same group. Therefore, in our experiment, an identical group of East Asian experimental participants evaluated the seven basic labels, including "surprise," for both the masked and non-masked conditions.

### Representation methods

The performance of multi-class classification models has often been evaluated using precision metrics or confusion matrices [13–15], which are useful for evaluating classifications in a straightforward manner. In general, these values and tables are generated by pairs of single labels per image, as shown by the majority data in Table 2. The vote count is the number of original votes by the ten FERPlus labelers. The upper face (#35320) in Table 2 has five votes for “anger,” two for “happiness,” and one each for “disgust,” “sadness,” and “surprise”; the majority method binarized that only "anger" is "True," and this technique is called one-hot encoding in the machine learning field. The multi-class classification network model has been evaluated using such one-to-one majority labels [13–15]. To train a network model based on one-to-one mapping of labels, minority votes after the second candidate were ignored in the results. Moreover, the vote rate can be expressed as [0.5, 0.1, 0.0, 0.2, 0.1, 0.1, 0.0] for the upper face (#35320) in Table 2. If such vector data are used as a label for the image, the variation in the original vote counts can be considered. In this study, we report on the results of both the majority and vote rate analyses.

**Table 2:** Three types of data representation methods for two non-masked faces.

Face ID	Method	Emotion labels						
		Anger	Disgust	Fear	Happiness	Sadness	Surprise	Neutral
#35320	Vote count	5	1	0	2	1	1	0
	Majority	**True**	False	False	False	False	False	False
	Vote rate	0.5	0.1	0	0.2	0.1	0.1	0
#34791	Vote count	0	0	5	1	0	4	0
	Majority	False	False	**True**	False	False	False	False
	Vote rate	0	0	0.5	0.1	0	0.4	0

*Note*. The vote count is the number of original votes by the ten FERPlus labelers.

In summary, FER is affected by the observer’s CB, the masking conditions of the target face, and the representation methods for the judgments. The CB difference or the mask shielding modifies how facial expression is categorized, with the shifts in precision likely depending on the original emotion label. To elucidate human FER and improve machine-learning-based FER, these effects need to be clarified. We have not found any detailed studies on the impact of both CBs and masks that focus on machine-learning dataset labels, considering evaluation methods. We conducted an FER experiment on the participants under masking and non-masking conditions and compared the results with the FERPlus labels for non-masked conditions, considering the differences in both voting schemes (majority or vote rate).

## Method

### Participants

We conducted an online FER questionnaire in which participants observed stimuli on their monitors and responded via a web form (Google Form). Seventeen volunteers (5 women and 12 men; age M = 27.5 years and SD = 9.9; 4 Chinese and 13 Japanese) comprising students or research associates of Yokohama National University participated in the online questionnaire after providing informed consent for the study participation. All participants were provided written and verbal consent prior to participation to the questionnaire in accordance with the Yokohama National University Rules on Life Science Research. This online protocol was exempted from ethical approval and conducted in accordance with the guidelines of the Yokohama National University Life Science Research’s “Decision Chart for Application for Review of Research Involving Human Subjects.” All information was gathered and analyzed anonymously. All experiments were performed according to the principles expressed in the Declaration of Helsinki.

Regarding the number of participants, the Western evaluator group, which is compared to the East Asian group, is based on the existing FERPlus research with a fixed sample size of 10 participants. Additionally, one of the objectives of this study is to investigate the characteristics of the FERPlus dataset, which is commonly used for training machine learning models. As a reference, previous research by Jack et al. [19] compared differences in the cultural backgrounds of FERs with a sample size of 13 Westerners and 13 East Asians. Given this context, we aimed to ensure a balanced comparison between culturally distinct groups while accommodating the availability of participants. Consequently, we independently chose a sample size of 17 East Asian participants, ensuring a comparable sample size to the fixed sample size of the Western group. We believe our chosen sample sizes offer a reasonable balance between maintaining consistency with previous research and ensuring an adequate sample size for our analysis of between-subject variability.

For simplicity, the FERPlus observers were described as "Western," and the participants in this experiment were described as "East-Asian." Thus, hereafter, "Western" and "East-Asian" represent all observers (evaluators) in their respective groups, with the target faces observed were common to both evaluator groups, primarily consisting of Western faces.

### Materials and procedure

**Fig 2:**
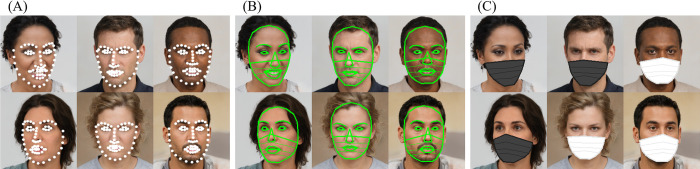
Examples of synthesized images and the online FER evaluation experiment using those images. (A) 68 facial landmarks were obtained. (B) Connections. (C) Images of synthesized masks. Reprinted from https://generated.photos under a CC BY license, with permission from the website’s owner, original copyright 2024. The images of the faces are examples for illustrative purposes only and are different from the original images used in the study.

The images of non-masked faces were randomly sampled from the FER2013 dataset. Fourteen faces were obtained from each of the seven emotion categories (anger, disgust, fear, happiness, sadness, surprise, and neutral), totaling 98 faces. These 98 faces were all different individuals’ facial photos. The mask images were generated from the original images of non-masked faces as follows. First, 68 facial landmarks were detected from each facial image using the DLIB face detector model [33] (Fig 2A). After connecting the necessary points from the obtained facial landmarks, the face in each image was divided into different regions (e.g., eyes, nose, mouth, contours, and eyebrows), as shown in Fig 2B. In addition to the 68 default landmark points, arbitrary points were obtained when calculating the interior and exterior points; infinite shapes of regions were generated. Six-type region sets of point clouds (3 wide types and 3 round types) were defined in the previous study for masked facial regions [34]. The coverage of this mask region corresponded to “wide medium” coverage in those regions. Regarding the mask color, a previous study [35] on mask color reported that there was no significant difference in the FER depending on the mask color (white or black), and that the FER recognition rate did not change significantly regardless of whether the color was black or white. Another study [36] also reported that the mask color only changed the FER rate slightly and did not show any systematic trend that would lead to a clear conclusion. Therefore, we synthesized a mask using computer graphics by randomly filling the knotted lower half of the facial region with white or black (Fig 2C). This process was implemented on Visual Studio Code (Microsoft) using Python and several libraries, such as DLIB and OpenCV.

**Fig 3:**
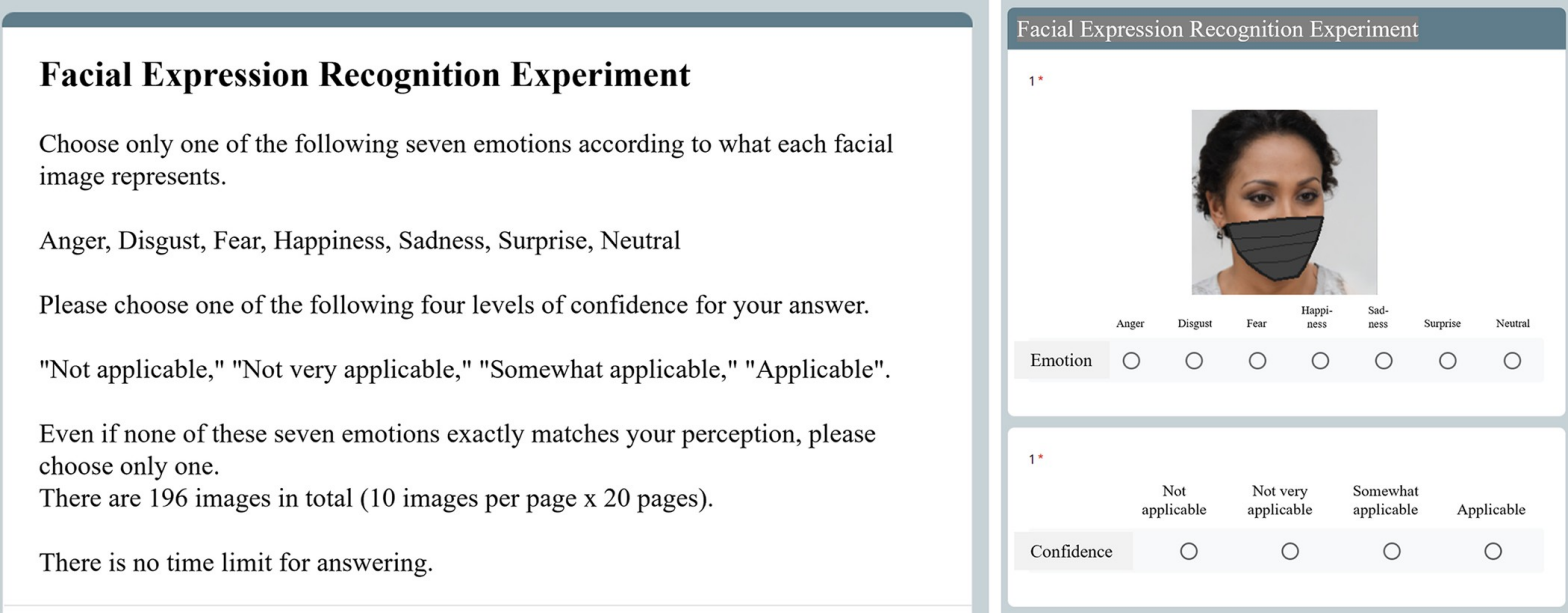
The online FER evaluation experiment. The experimental participants selected one of seven facial expressions (anger, disgust, fear, happiness, sadness, surprise, or neutral) for the presented images containing either masked or non-masked faces. Reprinted from https://generated.photos under a CC BY license, with permission from the website’s owner, original copyright 2024. The image of the face is an example for illustrative purposes only and is different from the original image used in the study.

The participants observed the stimuli presented on their monitors and responded on a web form (Google Form), as shown in Fig 3. The images of 98 masked faces that were generated from the original non-masked faces were presented in random order, followed by the 98 non-mask faces in a random order. For each face image presented in this order, participants were asked to identify the emotion on each face. Participants selected one of seven emotions for each presented face. They were also asked to select the level of confidence they had in their answer according to a four-point scale (not applicable, not very applicable, somewhat applicable, and applicable); these data were not used in the present analysis. No time limit was set for providing responses. All labeling could be completed in approximately 15 min, so it is believed that there was no extreme fatigue effect. Moreover, as the order was "masked (relatively difficult to evaluate) -> non-masked", such fatigue effects were minimal.

As FERPlus does not include data for "masked," we performed a direct comparison of "masked vs. non-masked" within a participant group in our experiment. As a result, the same observers could evaluate the originally identical target face with and without masks. Existing studies [21, 30] have not made this direct comparison because of the assumption that the labels in the FACES dataset [32] are considered unconditionally "True." As the masked images were generated from the same original non-masked facial image, if the two presentation orders "masked -> non-masked" and " non-masked -> masked" were randomly mixed, ambiguity could arise as to whether the original correct facial expressions had been consciously/unconsciously perceived. For this reason, we adopted a simple method in which the same observer first evaluated the relatively difficult masked condition and then the non-masked condition.

### Data analysis

The confusion matrices for the multi-class classification were created using the following procedure. The first raw data consisted of vote counts that were obtained from the present experiment using the FERPlus dataset, as shown in Table 2. We examined the majority and individual decisions to determine the differences in data representation. The majority decision was the one that received the most votes in each set per facial image. Regarding the duplication of the largest number of votes, the former was applied in the label order (anger, disgust, fear, happiness, sadness, surprise, and neutral). We derived 98 results for Westerners for non-masked faces, 98 results for East Asians for non-masked faces, and 98 results for East Asians for masked faces; results on Western (FERPlus) observers for masked faces were not obtained.

Regarding the individual judgments, 17 East Asians participated in the FER experiment; 1,666 (17 observers × 98 target faces) votes per condition were obtained. However, the 10 Western observers provided 980 (10 × 98) votes for non-masked faces. As the FERPlus dataset also provided the results with additional labels such as "contempt," "unknown," and "not found," the 930 decisions for the seven emotions were selected from the total 980 votes. Incidentally, every horizontal axis of the confusion matrices reported in this study was fixed to a majority decision and the vertical axis represented the individual or majority decision for each group. Each cell of the confusion matrix contained the probability (0.0 to 1.0) value that was calculated by dividing the number of votes counts by the number of observers. Thus, each column of each label totaled 1 when added vertically, although there were certain cases in which the sum was less than 1 because of the truncated floating value. The symbol *P* at the top of each confusion matrix represents the values of the precision index, which is calculated by the following formula:

P=TP/(TP+FP) (1)

where *TP* is true positive, and *FP* is false positive.

The *P* (precision) corresponds to the probability of matching labels between the two axes of the confusion matrix; that is, the ratio of right descending diagonals to the total element counts on the matrix layout. It is an index for the classification performance, which has been reported as the "facial expression recognition rate" that is commonly used in the fields of human [21–23] and machine-learning-based [13–15] FER. The value *K* above the confusion matrix represents Cohen’s Kappa coefficient [37], which accounts for the proportion of disagreements (off-diagonals in the confusion matrix), whereas *P* focuses on the agreements (diagonals); *K* is calculated as follows:

K=2(TP∙TN−FN∙FP)/{(TP+FP)∙(FP+TN)+(TP+FN)∙(FN+TN)} (2)

where *TN* is true negative, and *FN* is false negative.

## Results

### Confusion matrices

**Fig 4:**
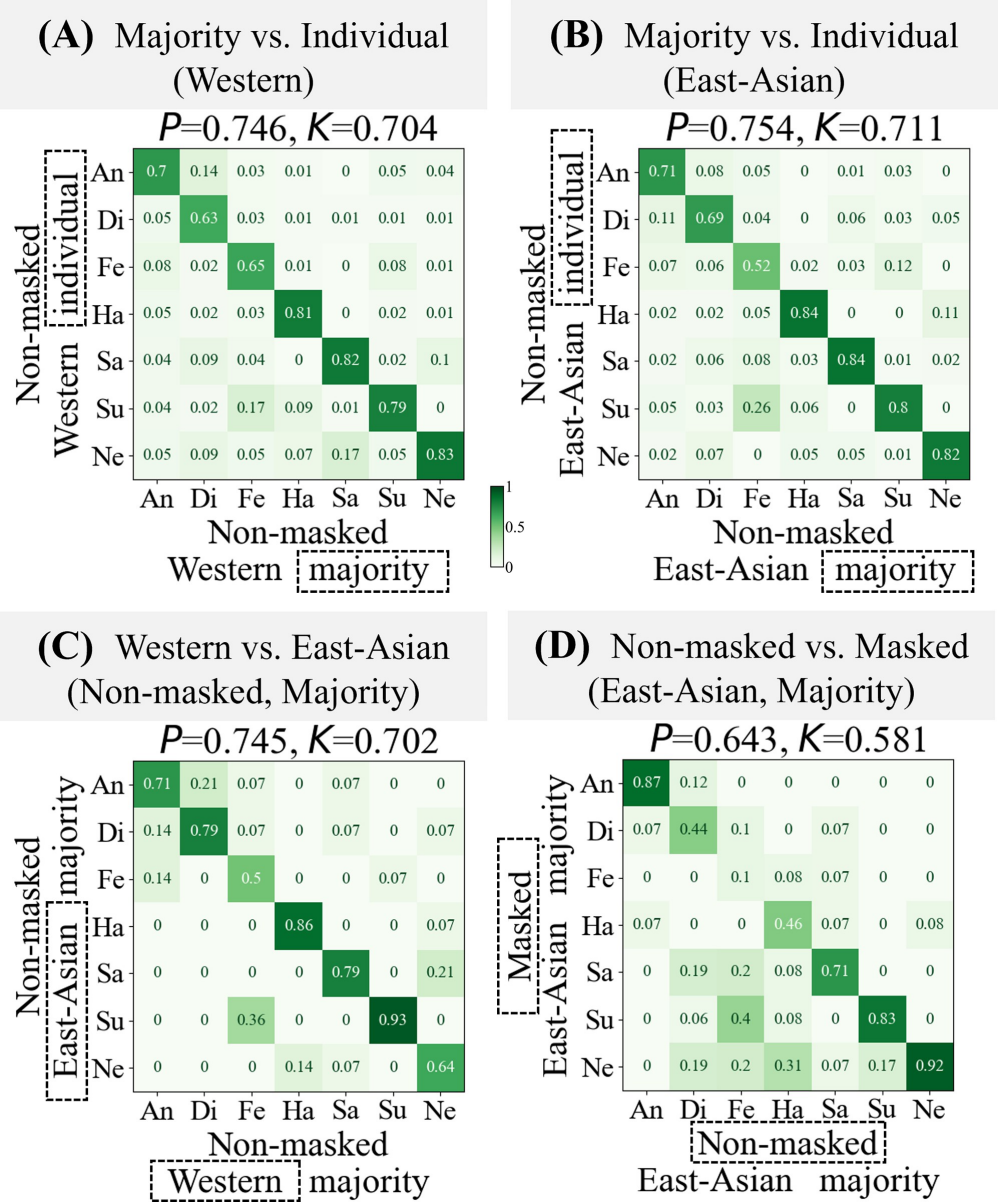
Confusion matrices comparing the judgments of observers according to different conditions. Only the condition in the dashed boxes differs between the vertical and horizontal axes. (A) Comparison of majority and individual perceptions of Westerners for non-masked target faces. (B) Comparison of majority and individual perceptions of East Asians for non-masked target faces. (C) Comparison of majority perceptions between Western and East Asians for non-masked target faces. (D) Comparison of majority perceptions of East Asians for non-masked and masked target faces.

The confusion matrices (Fig 4) were created from the evaluated emotion labels that were judged by the Western (FERPlus) and East-Asian (experimental participants) observers. The seven emotion labels in the confusion matrices were abbreviated as "An" (anger), "Di" (disgust), "Fe" (fear), "Ha" (happiness), "Sa" (sadness), "Su" (surprise), and "Ne" (neutral). In this section, we discuss the characteristics of the obtained confusion matrices.

#### Majority vs. individual (Western)

The matrix in Fig 4A shows how the judgments of individual Westerners are distributed compared to that of the majority. The coincident indices were the precision *P* = 0.746 and Cohen’s kappa coefficient [37] *K* = 0.704, implying that 20–30% of individual observers within an identical group responded differently from the majority. We performed a Fisher’s Exact test on the confusion matrix comparing the majority judgments to the individual judgments of Westerners. The top two significant differences, excluding the diagonal, were the frequency of individual Westerners judging "fear" as "surprise" (p = 2.20 × 10⁻⁷) and "sad" as "neutral" (p = 4.18 × 10⁻⁷), which were significantly higher than expected.

#### Majority vs. individual (East-Asian)

In the same manner, Fig 4B shows the distribution of the judgments of individual East Asians compared to that of the majority; *P* and *K* were 0.754 and 0.711, respectively, implying that 20–30% of East Asians also differed in their responses from that of the majority. The top two significant differences, excluding the diagonal, were the frequency of individual East Asians judging "fear" as "surprise" (p = 2.04 × 10⁻^13^) and "surprise" as "fear" (p = 3.40 × 10⁻^11^), which were significantly higher than expected.

Overall, "fear" judged by the majority was also judged as "surprise" by both Western (p = 2.20 × 10⁻⁷) and East-Asian (p = 2.04 × 10⁻^13^) individuals, indicating that, in addition to being judged as "fear," it was also recognized as "surprise."

#### Westerners vs. East Asians (non-masked, majority)

Fig 4C shows a comparison between the Westerners and East Asians. Both axes represent majority judgments for the non-masked faces. The coincident indices were *P* = 0.745 and *K* = 0.702. The only significant difference was the frequency of Westerners judging "fear" as "surprise" (p = 4.35 × 10⁻^2^), which was significantly higher than expected.

#### Non-masked vs. masked (East Asians, majority)

Fig 4D shows a comparison between the non-masked and masked target faces (*P* = 0.643, *K* = 0.581); both axes represent East-Asian majority judgments. For the individual vertical axis, *P* and *K* were 0.547 and 0.469, respectively. As the FER2013 and FERPlus datasets did not contain either images or labels for these masked faces, we compared them within the group of East Asians in this study. The top two differences were the frequency of non-masked faces judged as "fear" being judged as "surprise" for masked faces (p = 7.26 × 10⁻^2^) and non-masked faces judged as "happy" being judged as "neutral" for masked faces (p = 7.26 × 10⁻^2^).

In summary, through the above four types of comparisons, the most notable differences based on the observer’s cultural background and the masking conditions of the target face were related to “fear.” East Asians were more likely to perceive “fear,” judged by Westerners, as “fear” and additionally as “surprise”. Moreover, East Asians tended to perceive “fear” in non-masked faces as “fear” and additionally as “surprise” in masked faces.

#### Examples of differences in FER among observer groups

**Fig 5:**
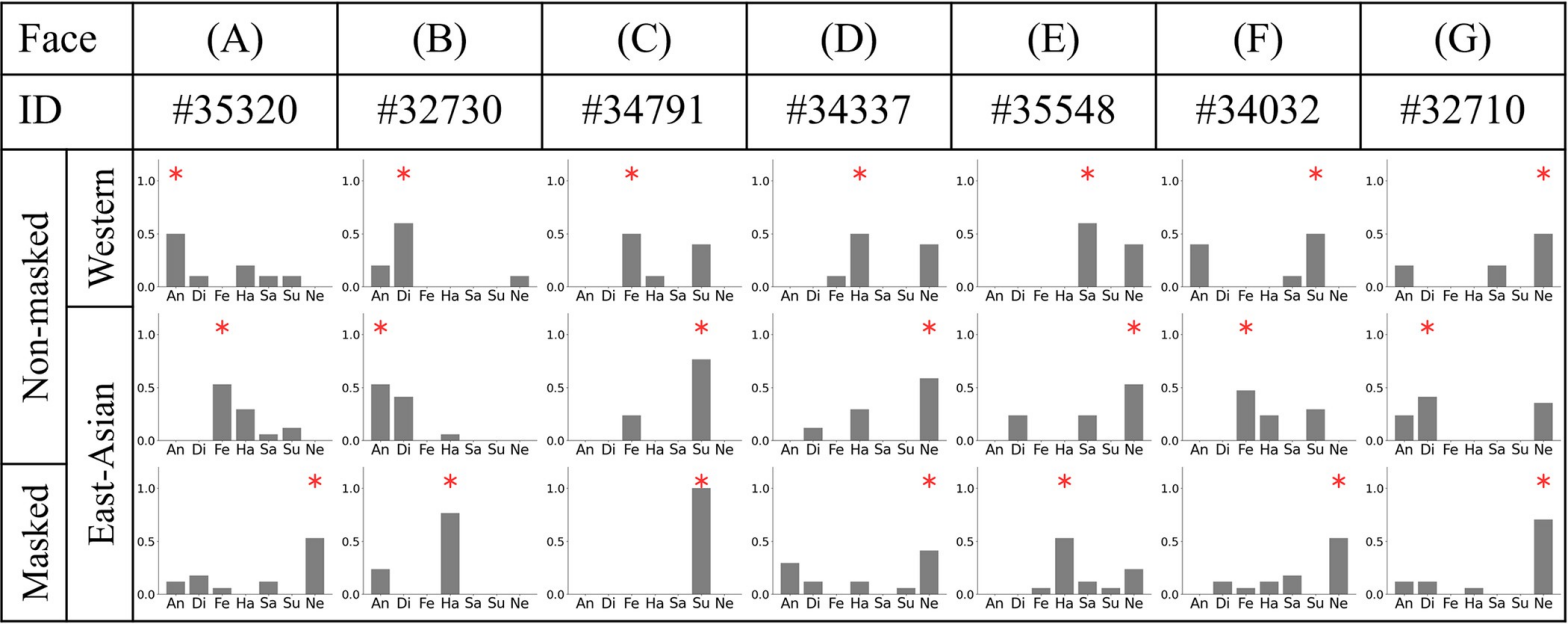
Examples of faces and vote rates for perception of emotions under different conditions. The red asterisk marks indicate the major judgments under each condition.

Fig 5 illustrates the differences in FER for certain examples according to different categories. The first, second, and third rows represent the vote rate distributions of Westerners for a non-masked face, East Asians for a non-masked face, and East Asians for a masked face, respectively. The red asterisk mark on the bar graph indicates the maximum value, which is the majority decision. As indicated in Fig 5A, the major decisions were “anger,” “fear,” and “neutral” from the top down; no East Asians perceived “anger” for the non-masked face. In Fig 5C, “fear” that was perceived by Westerners was perceived as “surprise” by East Asians; this was the most typical difference between the groups, as described in the previous section. In Fig 5G, “disgust” on the non-masked face perceived by East Asians was perceived as “neutral” by both Westerners for the non-masked face and East Asians for the masked face. That is, East Asians shifted from perceiving “disgust” for the non-masked face to “neutral” for the masked face, which was the same emotion that Westerners perceived for the non-masked face.

#### Differences between representation methods (majority/vote rate)

**Fig 6:**
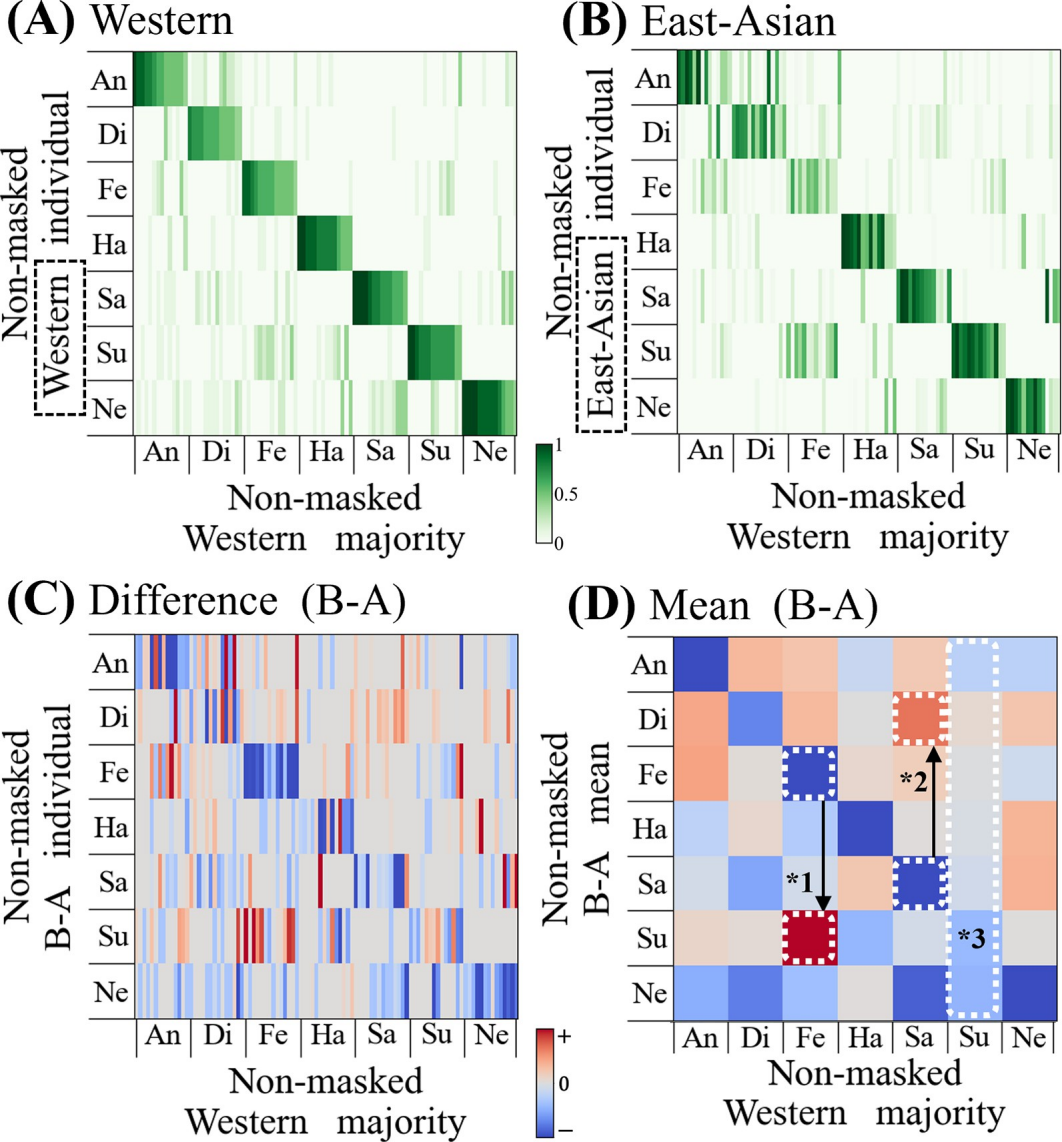
Heatmaps of vote rates of Westerners and East Asians. (A) Heatmap of vote rates of Westerners. (B) Heatmap of vote rates of East Asians. (C) Difference in heatmaps of Westerners and East Asians. (D) Averaged map of differences in heatmaps.

In the previous section, we compared the majority-based decisions of observers belonging to different observer groups or non-masked faces. The previous results (see Fig 4A and 4B) revealed that the observers tended to confuse “fear” and “surprise” (0.17: Western, 0.26: East-Asian), even within a single group. As mentioned in the Introduction, the majority method corresponds to one-hot encoding labeling in conventional one-to-one machine learning models. However, in the majority voting method, necessary information may be truncated in the binarization stage (see Table 2). In this section, we compare the majority and vote rate methods. The rates were calculated by dividing the number of votes cast by the number of voters in each group (see “Methods”). For clear extraction of the shifts in perception from the Westerners to East Asians, we calculated the difference between the Western and East-Asian vote rates for the same corresponding image. These calculations were expected to cancel any bias that originally existed within a group. Fig 6A and 6B show heatmaps for visualizing the image-by-image voting rate of Western and East-Asian. Each vertical thin bar in the heatmap represents the vote rate distribution for an image. For the correspondence of each facial image, the horizontal axis is arranged in the same order as that of the original labels; that is, the majority decision of Western. To consider the difference (B-A), we obtained the signed heatmap (C), which visualizes the differences between Westerners and East Asians. Fig 6D shows the averaged map of (C) for each paired category (7 x 7 elements). It presents the comparison in the shifts of judgments between East Asians and Westerners; darker red indicates a higher degree. An analysis of each element in Fig 6D reveals that there were significant differences in the perception shifts between East Asians and Westerners for two emotion pairs. Firstly, "fear" shifted to "surprise" (*1) more for East Asians than for Westerners, with a t-statistic of 3.79 (p < .05, using the Benjamini-Hochberg method for FDR correction), thereby agreeing with the results in the previous section (see Fig 4C). Secondly, the perception shifted from "sadness" to "disgust" (*2) with a t-statistic of 3.19 (p < .05, using the Benjamini-Hochberg method for FDR correction). In contrast, there were fewer differences between Westerners and East Asians regarding their perceptions of "surprise" (*3). The corrected p-values for "surprise" compared to other emotions were all above .05, indicating that no significant differences were detected: "surprise" vs "angry," "disgust," "fear," "happy," "sad," and "neutral" (p > .05). This lack of significant differences for "surprise" contrasts with the significant differences found in other emotion pairs. That is, the comparison of FERs between the Westerners and East Asians demonstrated that more detailed FER information can be represented when using the vote rate method than when using the majority method.

A Tukey–Kramer test was conducted on the mean value of the vote rates for each image. For the non-masked face, East Asians perceived “fear” significantly less than they did the other emotions (Ha, Sa, Su, Ne: p < .001, An: p < .05) compared to the Westerners. Furthermore, East Asians perceived “fear” on the masked face significantly less than they did the other emotions (Ha, Sa, Su, Ne: p < .001, Di: p < .001, An: p < .05) compared to their perceptions of the non-masked face; this was the same for "disgust" (Ha, Su: p < .001, Sa, Ne: p < .001). Overall, the rates of "fear" and "disgust" tended to be relatively lower compared with the other emotions, especially for masked faces.

**Fig 7:**
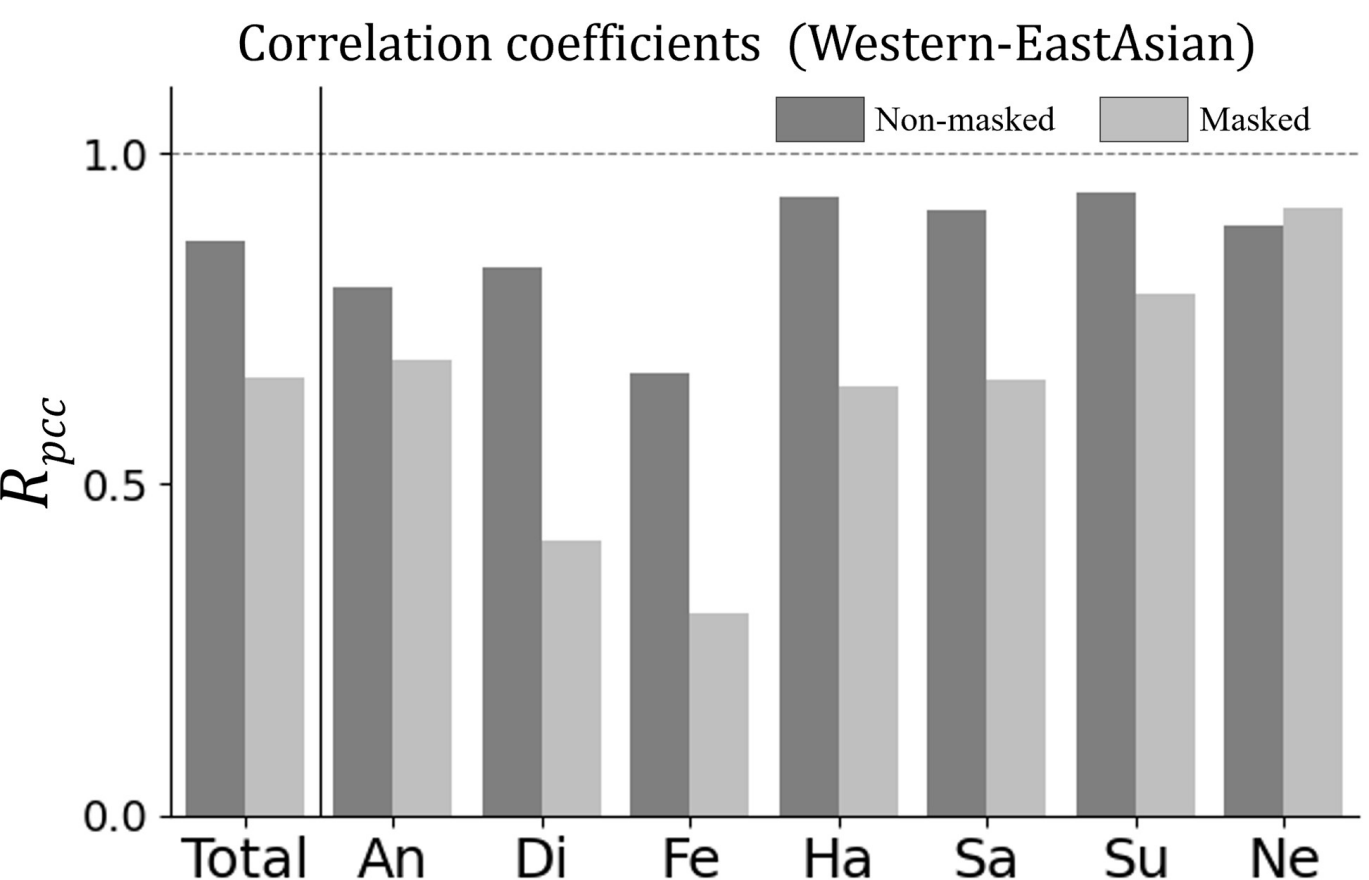
Correlation between Westerners and East Asians. This figure illustrates the correlation coefficients between the voting rates for each image by the Western (non-masked) and East Asian groups under both conditions, with and without masks. The correlation coefficient for "fear" was significantly lower than that for "happiness" and "surprise" (Fisher’s r-to-z transformation, p < .001).

Next, we calculated the correlation coefficients between the categorization rates of "Westerners (non-masked)" and "East Asians (non-masked)" as well as "Westerners (non-masked)" and "East Asians (masked)" and visualized these values in a bar graph for easier comparison (Fig 7).

As a result, for the non-masked face, the correlation between the categorization rates of "Westerners (non-masked)" and "East Asians (non-masked)" was relatively low for "fear," indicating a pronounced discrepancy in how this emotion was perceived. In contrast, "happiness" and "surprise" showed relatively high correlation coefficients. To verify these differences, we conducted Fisher’s r-to-z transformation to compare the correlation coefficients. The analysis revealed that the correlation for "fear" was significantly lower than that for "happiness" and "surprise" (p < .001), indicating a more pronounced discrepancy in how "fear" was perceived between "Westerners (non-masked)" and "East Asians (non-masked)." Similarly, for the masked face, the correlation between "Westerners (non-masked)" and "East Asians (masked)" was also found to be significantly lower for "fear" than for "happiness" and "surprise" (p < .001). However, the interpretation of these correlation coefficients should be approached with caution due to the absence of masked data for the Western group.

Additionally, to better understand the agreement between the groups, we also calculated the average Cohen’s kappa values [37, 38], a more appropriate measure for assessing agreement between two groups. Cohen’s kappa coefficient is a statistic that measures inter-rater agreement for categorical items. The average Cohen’s kappa value for the non-masked face condition was 0.299, and for the masked face condition, it was 0.209. These relatively low kappa values indicate overall differences in how facial expressions are perceived by Westerners (non-masked) and East Asians (both non-masked and masked), further supporting the results observed in the previous sections. However, like the correlation coefficients, the interpretation of the kappa values for the masked face condition should be approached with caution due to the lack of masked data for the Western group.

**Fig 8:**
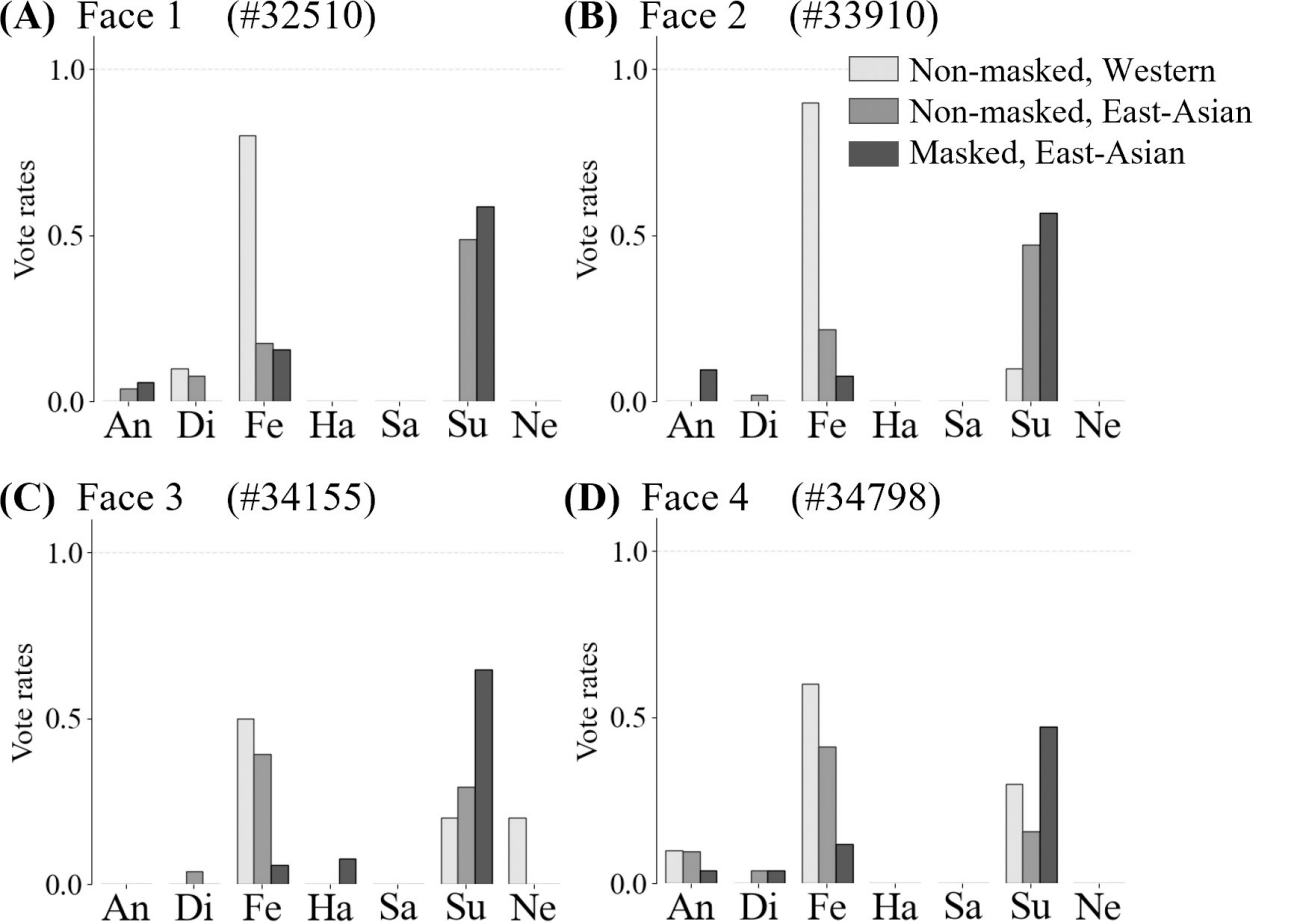
Examples of faces and vote rates. It primarily shows the relatively low correlation coefficients between the voting rates for "fear" by Westerners and East Asians (p < .001).

This analysis indicated that the most substantial changes in perception among the different cultural backgrounds of observers and masking conditions were for "fear." Fig 8 shows four examples of the large differences in perceptions of "fear" under different conditions. Fig 8A and 8B show that the "fear" perceived by Westerners for the non-masked face was perceived as "surprise" by East Asians for both the non-masked and masked faces. Additionally, Fig 8C and 8D illustrate that the "fear" perceived by both Westerners and East Asians for the non-masked face was perceived as "surprise" by the East Asians for the masked face.

The observation of the "fear"-"surprise" perceptions on the confused faces (see Fig 8) inspired the hypothesis that the opening size of the eyes on the target face may be related to the perceptions of "fear" and "surprise" by the observers; this is examined in a later section.

#### Projection map of observer vote rates

**Fig 9:**
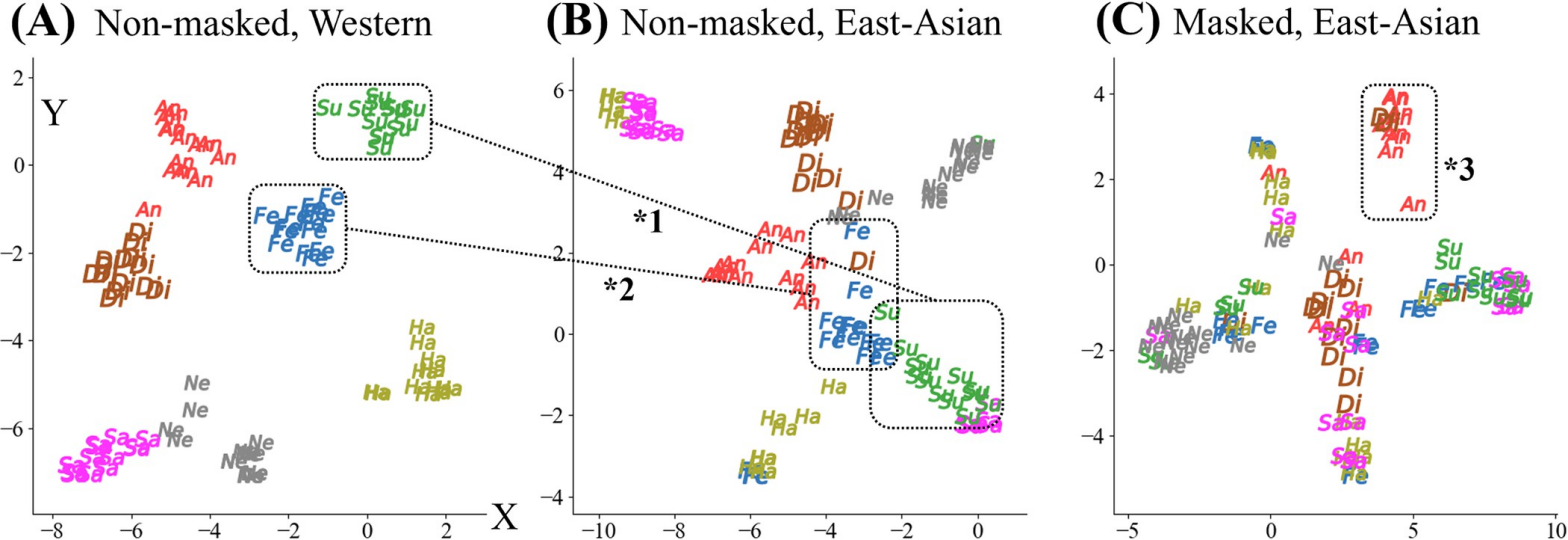
Comparison of t-SNE projection. Projection maps of observer’s vote rates using dimensionality reduction technique t-Distributed Stochastic Neighbor Embedding (t-SNE) [39].

Fig 9A–9C depict the projection maps of the participant voting rates (7-dimensional vector), which were dimensionally reduced by t-distributed stochastic neighbor embedding (t-SNE) [39]. The 7-dimensional vectors represent the voting rates for each of the seven facial expressions. The t-SNE technique is a machine learning algorithm used for dimensionality reduction, particularly well-suited for visualizing high-dimensional data in a lower-dimensional space. It works by converting similarities between data points to joint probabilities and then minimizing the divergence between these probabilities in the original high-dimensional space and the reduced lower-dimensional space. This allows us to observe patterns and relationships in the data that are not easily visible in the original space.

For our data, these figures visually indicate whether the participant responses were similar (close in the scatter plots) or dissimilar (distant). Additionally, a relative shift in the ease of identification could be observed under the same reductional conditions. In the case of Western perceptions for non-masked faces (Fig 9A), the labels were relatively distant, indicating the relative ease of these observers in discriminating different emotions. In the case of East-Asian perceptions for non-masked faces (Fig 9B), "fear" and "surprise" were relatively close and overlapped (*1 and *2); "fear" shifted to the center around position (*2), which also overlapped with "anger" or "disgust," and "surprise" shifted to the lower right (*1), remaining relatively independent from the other emotions. In the case of East-Asian perceptions for masked faces (Fig 9C), all labels overlapped, with "anger" in the top right corner, indicating that these observers found it relatively easy to discriminate "anger" from the other emotions, even for a masked face.

#### Opening rate of eyes for "fear" and "surprise"

East Asians tended to recognize the "fear" perceived by Westerners as "surprise" and had more difficulty discriminating "fear" from the other emotions than Westerners did. As described in the previous section, when observing a set of facial images conveying "fear" and "surprise," the eyes on the faces of the targets seemed to open relatively wider than they did for other emotions. Therefore, we assumed that the observers used the eye-opening rate as a visual cue when perceiving emotions, particularly for these emotions. We investigated the relationship between the evaluation labels and eye-opening rate for "fear" and "surprise." The eye-opening rates were obtained by the coordinates (*p*_0_ to *p*_5_) of six eye points from 68 facial landmarks [33], which had already been acquired when the CG mask was overlayed (see “Methods”). The following formula proposed by Tereza and Jan [40] was used to calculate the eye-opening rate *R*_*rate*_ (modified for adjustment in the range of 0.0 to 1.0):

$$R_{raw}=\frac{\left\|p_{5}-p_{1}\|+\|p_{4}-p_{2}\right\|}{2\|p_{3}-p_{0}\|},R_{rate}=(R_{raw}-0.15)*3$$ (3)

Note that the above index *R*_*raw*_, on which we focus in this case, is not the absolute size of the eye or its size relative to the face contour, but is, in effect, a rate corresponding to the aspect ratio of the eye (the denominator of Eq 3 is the width and the numerator is the height in two places of the eye).

**Fig 10:**
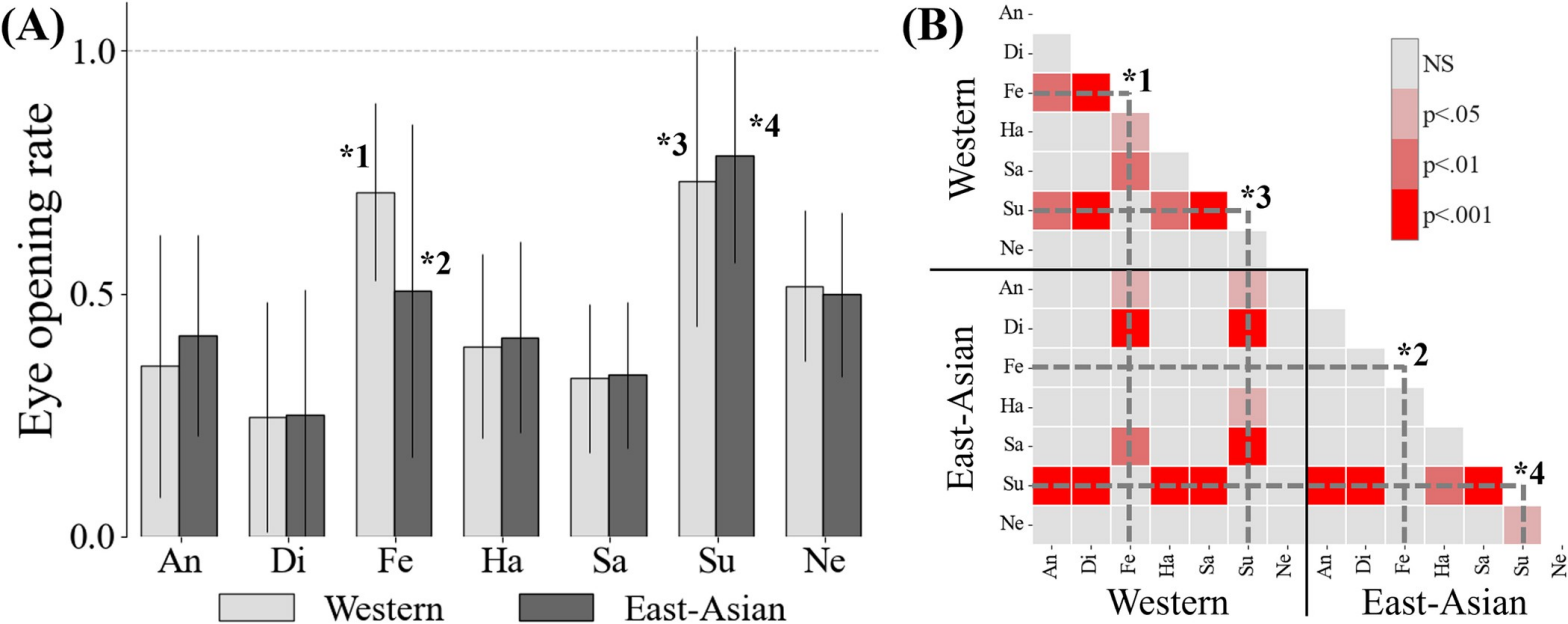
Eye-opening rate between different conditions. (A) Eye-opening rate of target faces for each label. The eye-opening rate is, in effect, a rate corresponding to the aspect ratio of the eye (See Eq 3). (B) P-value map of multiple Tukey–Kramer tests.

The mean value and standard deviation of the eye-opening rate of the image groups within each label are shown in Fig 10A and the p-value map of the multiple Tukey–Kramer tests is presented in Fig 10B. The p-value map indicates that "fear" for Western (*1) and "surprise" for Western (*3) and East-Asian (*4) were relatively higher than the other emotions (p < .001 and p < .01), which suggests that Westerners were more likely to recognize emotions on faces with large eyes as "fear" or "surprise." However, for the East Asians, "fear" was not significantly different from any of the other emotions (*2), suggesting that these observers were more likely to recognize emotions on faces with large eyes as "surprise" than "fear."

## Discussion

### Cultural background differences of observers

The results between Western and East-Asian (Fig 4C) show that *P* was 0.745 and *K* was 0.702; these represent the degree of coincidence between the observer groups in perceiving emotions on non-masked faces (see Fig 4C). These results indicated that 20–30% of judgments differed based on CB, supporting the results of previous studies [16–18, 20]. Our experiment revealed that "fear" perceived on target faces by Western tended to be perceived as "surprise" by East-Asian. Western tended to confuse "sadness" as "neutral" (0.21), whereas East-Asian did so relatively less (0.07). The vote rate analysis revealed that overall, "sadness" perceived by Western was perceived as "disgust" by East-Asian (see Fig 4D).

### Masking condition of the target face

With respect to the results of the masking conditions (Fig 4D), *P* was 0.547 and *K* was 0.469 when perceiving emotions on masked faces. The decrease in the precision from non-masked to masked faces (75.4%-54.7% = 20.7%) for East-Asian observers supports the results of previous studies [29, 31], which have reported that the precision of FER decreased for masked faces. This assessment revealed that the "fear" perceived for non-masked faces tended to be confused with "surprise," "sadness," or "neutral" for masked faces. In the evaluation group in this experiment, mask shielding changed "fear" labels to "surprise" labels across categories, which is not simply a decrease in the recognition rate or relative ease of confusion, as reported in previous studies [21, 29–31]. The "disgust" perceived for non-masked faces was confused with "sadness," "neutral," or "anger" for masked faces, and "happiness" for non-masked faces was confused with "neutral" for masked faces. These results partially support the report by Carbon [30] that states that for masked faces, "happiness" is more likely to be perceived as "neutral" and "disgust" as "anger." However, they also reported that their evaluation group tended to perceive "sadness" as "neutral" for masked faces, whereas our results showed that the East Asians in our study did not display such confusion (see Fig 4D). Therefore, we can infer that there is a CB-specific tendency to confuse emotions, rather than a CB-common one for masked faces.

### Representation methods

As reported in this study, FER results may be susceptible to the CB difference or masking condition; therefore, the differences in judgment by certain groups (e.g., those with relatively small numbers) should not be immediately dismissed as incorrect. In addition to the differences in the FER between CBs, the differences were owing to the manner in which the data were represented (majority/vote rate), especially when the dataset labels were used for machine learning. A majority voting method (one-hot multi-level classification) is generally employed in machine-learning-based FER training [13–15]. In the majority voting method, only the facial expression that receives the highest number of votes from the group of judges is used as the facial expression label for that face image (see Table 2). We presented and compared the results of both the majority vote and vote rate methods to investigate whether there are any noteworthy differences in the representation of FER between these methods. The results demonstrated that the vote rate method exhibited a change in cognition from sadness to disgust, whereas the majority voting method did not (see Figs 4C and 6D). Thus, it is believed that the majority voting removed necessary information (after the second candidate for a certain facial expression). The vote rate maps that were dimensionally reduced (see Fig 9C) indicated that "anger" was relatively isolated from the other labels, suggesting that East-Asian were more sensitive to negative facial expressions such as "sadness" or "anger." These results suggest that, especially for ambiguous expressions that tend to differ among CB or masking conditions, it may be desirable to adopt the vote rate method instead of the majority vote method to provide more detailed classification.

### Eye opening rate

Our analysis of the eye-opening rate revealed that East-Asian were more likely to perceive emotions on faces with widely opened eyes as "surprise," whereas Western recognized them as either "surprise" or "fear" (see Fig 10A), suggesting that "fear" was relatively ambiguous and easily influenced by the masking condition of the target face or difference between evaluation groups. A previous study [19] conducted an FER evaluation experiment with 13 Westerners and 13 East Asians, focusing on the eye movements of evaluators during FER, and found that East Asians tended to focus on the eye region during emotion recognition compared to Westerners. Furthermore, East Asians tended to confuse "fear" and "surprise" because the eye regions of the "fear" and "surprise" expressions are similar. Our results support these findings and show not only confusion, but a category reversal, with more responses to the "surprise" label than the "fear" label. Another study [41] conducted an FER evaluation experiment with German university students to investigate which regions of the target face contributed to the judgment and found that the focus was on the mouth region for "disgust" and "happiness" but on the eye region for "fear" and "sadness." Eyelid lifting made a significant contribution to "fear," and both the eye and mouth regions were involved in "surprise." In our study focusing on the eye-opening/closing rate, the eye-opening ratio predicted "surprise" well for both Western and East Asian observers. However, for "fear," the eye-opening ratio did not predict fear as well for East Asians as it did for Westerners. On the other hand, Jack et al.’s research [19] has shown that Westerners and East Asians use different facial features to interpret the same emotional expressions. Specifically, Western Caucasians predominantly rely on the eyebrows and mouth, while East Asians show a preference for expressive information in the eye region. This result suggests that East Asians and Westerners may have different expectations about changes in the eye area during a fearful expression. Overall, these findings highlight the importance of considering cultural differences in facial expression recognition and suggest that tailored approaches may be necessary for accurate emotion detection across different cultural groups.

### Application to the machine-learning FER

Regarding the dataset for the general object recognition of machine learning, "bicycles" of various types, postures, and shielding were assigned a single label of "bicycle," and different types of "traffic lights" were assigned an aggregated label of "traffic light." Such objects would be perceived similarly even by people from different cultural backgrounds because they are relatively less ambiguous than facial expressions. In contrast, labels for facial expressions are assumed to be determined by subtle cues such as the inclination or distortion of the eyes, nose, mouth, or contours. The recognized emotion even differs depending on whether the observer perceives whether the eyes of the target are opening as widely as they normally do or more widely than normal, implying the nuances that are involved in these judgments. The present report showed that masking changed how emotions were perceived. This shift was observed in the East-Asian observer group, indicating that emotion recognition may vary depending on the visual cues (e.g., eyes and mouth) that observers focus on (see section on eye-opening rate). Therefore, it is desirable to adopt the estimation model carefully according to the situation (e.g., the CB of the evaluation group, masked or non-masked faces) in machine-learning-based FER. Extending the training dataset according to the situation and transferring learning data is one of the reasonable solutions. However, a sufficiently large dataset of masked faces and shifted labels is not always available for each group. Thus, in such a case, the prediction should be adjusted according to the results, based on the shift in perceptions for the simulations.

### Future works

In this report, we focused on comparing existing machine-learning datasets (evaluated by Westerners) and thus did not include evaluation labels for masked faces by Westerners. However, future research should include evaluations of masked faces by Westerners to fully understand the interaction effects. Additionally, the differences in the target face group (e.g., cultural background) should also be considered for future developments in FER. In this study, we used the existing datasets FER2013 [2] and FERPlus [3] for application to machine-learning-based FER. These datasets also contain some East Asian facial images, such as the face in Fig 5G (#32710). The recognized labels on the image show that Western and East-Asian recognized the emotions differently as “neutral” and “disgust," respectively. However, for the masked face, both perceived the emotion as "neutral." Previous studies [42–44] have shown that the recognition of faces or emotions was most accurate when the observer and target belonged to the same CB. Accordingly, we can infer that Western struggled to perceive "disgust" on unfamiliar East-Asian target faces, whereas East-Asian perceived it more easily [42–44] based on information such as minute distortions near the mouth area of the face. When the mouth of the target was hidden by a mask, East-Asian struggled to perceive “disgust” and shifted their perception to “neutral,” which was perceived by Western. Accordingly, the relationship between the CBs of the observer and target needs to be investigated further in future studies.

Additionally, in this study, we primarily focused on exploring the diversity of human observers in categorizing facial expressions, particularly in the context of cultural differences and the impact of masking. However, based on our findings, comparing the accuracy and performance of machine learning classifiers directly with human observers will also be an important task for future research. This will be essential in ensuring the robustness and generalizability of automated FER systems.

## Conclusions

We conducted a facial expression recognition (FER) experiment using the machine-learning datasets FER2013 / FERPlus, with images of both masked and non-masked target faces. Our study compared the expression recognition data from different evaluation groups (Western vs. East-Asian). Firstly, the majority vote confusion matrix analysis revealed that "fear" judged by Westerners was often perceived as "surprise" by East Asians, and this was statistically significant (p < .001). Additionally, "fear" in non-masked faces was often perceived as "surprise" in masked faces across categories, suggesting a notable shift rather than simply lower recognition rates or confusion as in existing studies [21, 30]. Secondly, the detailed vote rate analysis indicated that East Asians were more likely to perceive "sadness" as "disgust" (p < .05), a finding that was not evident in the majority vote analysis. This highlights the importance of using the voting rate method for capturing subtle differences in emotion perception. Thirdly, the correlation coefficient analysis showed that the correlation for "fear" was significantly lower than that for "happiness" and "surprise" (p < .001), indicating a pronounced discrepancy in how "fear" was perceived between Westerners and East Asians. This was true for both masked and non-masked conditions, although caution is advised in interpreting these results due to the absence of masked data for the Western group. Additionally, the average Cohen’s kappa values were calculated, showing values of 0.299 for non-masked faces and 0.209 for masked faces. These values further support the observed differences in facial expression perception between the groups. Our analysis of the eye-opening rate suggested that Westerners were more likely to recognize emotions on faces with widely opened eyes as "fear" or "surprise," whereas East Asians mainly recognized them as "surprise." This indicates different expectations about eye changes during emotional expressions between the groups. These findings emphasize the need to consider cultural differences and masking conditions when using datasets for machine-learning-based FER. This insight can contribute to the development of more accurate and culturally sensitive facial expression recognition systems.

## Supporting information

S1 Dataset Experiment data. (ZIP)
