# Supplementary material for: Effect of observer’s cultural background and masking condition of target face on facial expression recognition for machine-learning dataset
Source: PLoS One. 2024 Oct 30;19(10):e0313029. doi: 10.1371/journal.pone.0313029 (PMC11524507; doi:10.1371/journal.pone.0313029)
Supplement: S1 Dataset — (ZIP) [file pone.0313029.s001.zip › Experiment Data/README.pdf]

Experiment Data Description

This README file provides a description of the experiment data files included in this submission.

File: `exp01.csv`

- **Description**: This file contains the correspondence of experiment stimuli numbers, presentation order, file names, and other information.
- **Columns**:
  1. **Trial Number**: Sequential number of each trial.
  2. **Random Order**: Randomized order of presentation for each stimulus.
  3. **Image File Number (Sequential)**: File name of the experiment image, numbered sequentially.
  4. **Image File Number (Random)**: File name of the experiment image, numbered according to the random presentation order.
  5. **Default Category (FERPlus)**: Default category of the image according to FERPlus.
- **Data Organization**: The first 98 rows correspond to the 'Masked' condition, and the following 98 rows correspond to the 'Non-masked' condition. The presentation order is randomized within each category, with 'Masked' presented first, followed by 'Non-masked'.

File: `Western/fer_fn_pair.csv`

- **Description**: A file describing categories and numbers for the facial image machine learning training datasets available from the FER2013 [2] and FERPlus [3] websites.
- **References**:
  - [2] Carrier PL, Courville A, Goodfellow IJ, Mirza M, Bengio Y. FER-2013 face database. Universit de Montreal. 2013.
  - [3] Barsoum E, Zhang C, Ferrer CC, Zhang Z. Training deep networks for facial expression recognition with crowd-sourced label distribution. Proceedings of the 18th ACM International Conference on Multimodal Interaction, 2016; 279-283.
- **Columns**:
  1. **Category**: The category (train/valid/test) as defined in FER2013 (common to FERPlus).
  2. **Original Emotion Label**: The original emotion label from FER2013.
  3. **Sequential Number**: Sequential number within each category (train/valid/test).
  4. **FER2013 ID**: The ID from FER2013 (common to FERPlus).

File: `Western/label.csv`

- **Description**: Raw voting data from 10 raters of FERPlus for each face image in the 'test' category.
- **Columns**:
  1. **Category (test) ID**: The ID in the 'test' category.
  2. **Facial Region**: Represents the facial region in the image, not used.
  3. **Votes**: Voting numbers from the 10 raters of FERPlus for each image. From left to right, the labels are Neutral, Happy, Surprise, Sad, Angry, Disgust, Fear, Contempt, Unknown, NotFound.

File: `EastAsian/emorec_exp_results.csv`

- **Description**: Raw response data from 17 East Asian participants in the experiment (refer to the 'Materials and Procedure' section of the paper).
- **Columns**:
  1. **Participant Simbols**: Anonymized symbols (A-Q) for the 17 experiment participants.
  2. **Response Data**: Repeated pairs of "Emotion" and "Confidence" for each trial, aligned with the trial numbers in `exp01.csv`.
- **Data Organization**: The first 98 items correspond to responses for the 'Masked' condition, and the following 98 items correspond to the 'Non-masked' condition.
